# Adult Stem Cells for Bone Regeneration and Repair

**DOI:** 10.3389/fcell.2019.00268

**Published:** 2019-11-12

**Authors:** Maria Rosa Iaquinta, Elisa Mazzoni, Ilaria Bononi, John Charles Rotondo, Chiara Mazziotta, Monica Montesi, Simone Sprio, Anna Tampieri, Mauro Tognon, Fernanda Martini

**Affiliations:** ^1^Department of Morphology, Surgery, and Experimental Medicine, University of Ferrara, Ferrara, Italy; ^2^Institute of Science and Technology for Ceramics, National Research Council, Faenza, Italy

**Keywords:** stem cell, regenerative medicine, differentiation, bone, repair

## Abstract

The regeneration of bone fractures, resulting from trauma, osteoporosis or tumors, is a major problem in our super-aging society. Bone regeneration is one of the main topics of concern in regenerative medicine. In recent years, stem cells have been employed in regenerative medicine with interesting results due to their self-renewal and differentiation capacity. Moreover, stem cells are able to secrete bioactive molecules and regulate the behavior of other cells in different host tissues. Bone regeneration process may improve effectively and rapidly when stem cells are used. To this purpose, stem cells are often employed with biomaterials/scaffolds and growth factors to accelerate bone healing at the fracture site. Briefly, this review will describe bone structure and the osteogenic differentiation of stem cells. In addition, the role of mesenchymal stem cells for bone repair/regrowth in the tissue engineering field and their recent progress in clinical applications will be discussed.

## Introduction

Bone disorders are seen on a daily basis in clinical management, with remarkable health, social and economic outcomes (Figliomeni et al., 2018). Annually, more than 20 million individuals are affected by loss of bone tissue (Habibovic, 2017). Bone repair after fracture is a complex process that leads to new bone formation through sequential cellular and molecular events regulated by systemic and local factors (Arvidson et al., 2011).

Although bone fracture repair usually restores the damaged skeletal organ to its pre-injury status, about 10% of fractures will not heal normally (Einhorn and Gerstenfeld, 2015). Indeed, in some cases, the bone regeneration process could fail in extensive bone resections due to osteosarcoma, osteoporosis, osteomalacia, osteomyelitis, avascular necrosis, and atrophic non-union (Gao et al., 2014; Ferracini et al., 2018).

In particular, osteosarcoma and Ewing sarcoma are the two most common types of bone cancers diagnosed in young subjects (Ward et al., 2014). Indeed, unlike other tumors which usually affect elderly people (Tognon et al., 2015; Mazzoni et al., 2016, 2017b; Rotondo et al., 2016, 2018), osteosarcoma and Ewing sarcoma are mainly diagnosed in children/adolescents and young adults with a prevalence of 56 and 33%, respectively (Ward et al., 2014). Current osteosarcoma treatment includes surgical resection in association with chemotherapy (Harrison et al., 2018). On the other hand, osteoporosis is a chronic disease that leads patients to an increased risk of developing fractures (Schumacher et al., 2013). This pathology is characterized by high morbidity and mortality in aging populations (Migliaccio et al., 2017). Affected bones can be restored to normal conditions in clinical practice using bone grafts, such as auto-grafts, allo-grafts, or xeno-grafts (Rasch et al., 2019). Autologous grafts represent the clinical gold standard (Cypher and Grossman, 1996) in improving bone regeneration due to perfect histocompatibilty, as well as osteoinductive and osteoconductive proprieties. However, auto-grafts still show some disadvantages resulting from the limited amount of bone available for grafting and donor site morbidity. Conversely, allo-grafts and xeno-grafts represent an alternative approach to bone grafts as they solve the problem of limited autologous bone supply and do not require an additional surgical site for graft harvesting (Delloye et al., 2007). However, allo- and xeno-grafts present some drawbacks, such as donor scarcity, high costs, infectious agent transmission risk or immune reactions (Ferracini et al., 2018; Ho-Shui-Ling et al., 2018). For these reasons, a more efficient clinical therapeutic strategy is needed. To this end, tissue engineering has employed new osteoconductive and osteoinductive biomaterials/scaffolds, stem cells, and growth factors to improve bone repair/regrowth (Iaquinta et al., 2019). Stem cells, in particular MSCs, are characterized by sustained self-renewal and expansion, multi-potentiality, anti-inflammatory and immune-modulatory effects, in addition to the secretion of molecules that can start or support tissue regeneration/substitution (Caplan and Dennis, 2006). Despite having been used in clinical applications for more than 20 years, the characteristics and potential for bone repair of stem cells are yet to be fully elucidated (Jin and Lee, 2018). Specifically, stem cells have been considered in several medical fields to repair defective tissues and organs including bone, ligament and the heart (Abdel Meguid et al., 2018). Thus, this review will focus on potential applications for stem cells, in particular MSCs, to improve the regeneration of bone tissue.

## Bone Structure

Bone is a rigid and highly dynamic tissue that supports and protects several organs in the body. Moreover, bone tissue provides the environment for red and white blood cell production, plays an important role in mineral homeostasis, such as calcium and phosphorus, and gives a solid base for skeletal muscles (Abdel Meguid et al., 2018). Two types of osseous tissue can be identified: solid cortical or compact bone, which represents 80% of bone mass, and trabecular bone, the remainder (Vico et al., 2017). Cortical bone is the hard-outer layer of the bone, while trabecular bone architecture is organized to optimize load transfer (Bayraktar et al., 2004). Trabecular bone can be found at the end of long bones, as well as in pelvic bones, the skull, ribs, and vertebrae. Furthermore, it contains red bone marrow where hematopoiesis takes place (Gdyczynski et al., 2014; Abdel Meguid et al., 2018).

**FIGURE 1:**
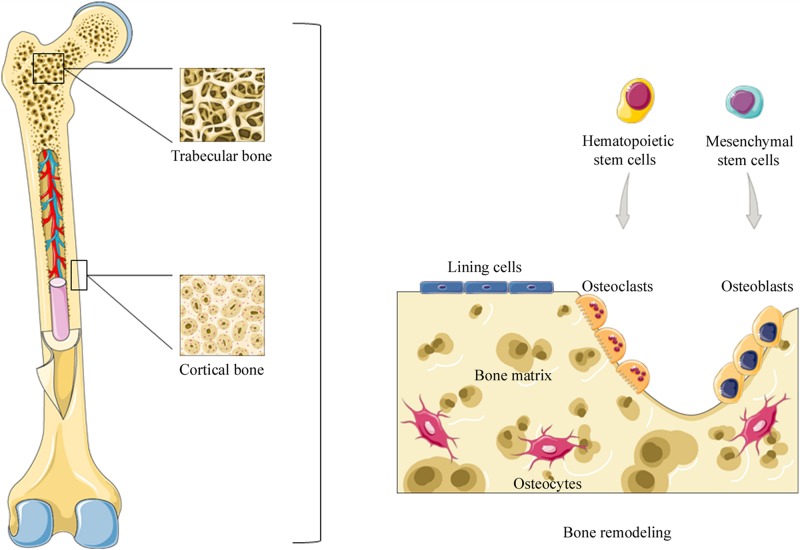
Representation of bone structure. Two types of osseous tissue can be identified: compact bone and trabecular bone. Bone tissue is subjected to bone remodeling, a life-dominant process that plays an important role in bone mass balance and mineral homeostasis. During bone remodeling osteoclasts, derived from hematopoietic stem cells, resorb old, or damaged bone. Subsequently, osteoblasts, derived from mesenchymal stem cells, are recruited to the damaged area in order to replace bone removed by osteoclasts. Instead, osteocytes derived from osteoblasts suspend their activity when buried in the bone matrix.

Cortical and trabecular bones are subjected to bone remodeling (see below), a life-dominant process that plays an important role in bone mass balance and mineral homeostasis (Tolar et al., 2004). Moreover, two different phases can be distinguished in bone tissue (i) bone matrix and (ii) an organic phase that includes cellular elements, such as osteoblasts, osteoclasts, and osteocytes (Farbod et al., 2014) (Figure 1).

### Bone Matrix

Bone matrix is a dynamic network that represents the intercellular substance of bone tissue. It is made up of several organic and inorganic components, such as collagen type I, which is the most abundant protein in bone tissue, elastin, polysaccharides, and calcium phosphate (Schönherr and Hausser, 2000; Fujisawa and Tamura, 2012; Farbod et al., 2014). The principal non-collagenous proteins of bone matrix are sialoprotein, osteonectin, osteopontin, and osteocalcin (Palmer et al., 2008), which contain aspartic acid (Asp) and glutamicacid (Glu) residues, with a high affinity for calcium ions (Ca^2+^) due to their charged carboxyl groups (Palmer et al., 2008). The polyglutamic acid segments in bone sialoprotein are responsible for binding the protein to apatite, while in osteopontin the same role is performed by polyaspartic acid segments (Ganss et al., 1999; Fantner et al., 2007). Osteonectin, a protein rich in cysteine amino acid, is expressed at a high concentration in mineralized tissues. Osteonectin is involved in osteoblast differentiation and osteoclast activity (Rosset and Bradshaw, 2016). Osteocalcin, also known as bone γ-carboxyglutamate protein (BGLAP), is expressed by osteoblasts and is commonly used as a clinical marker of bone turnover (Lambert et al., 2016). Bone matrix can regulate cell proliferation and differentiation through soluble growth factors and cytokines (Rosso et al., 2004). On the other hand, the inorganic component of bone matrix is an ion reservoir (Weatherholt et al., 2012). Hydroxylapatite [HA; Ca_10_(PO_4_)_6_(OH)_2_] is the most abundant inorganic crystal phase, containing citrate, carbonate and ions such as F^–^, K^+^, Sr^2+^, Pb^2+^, Zn^2+^, Cu^2+^, and Fe^2+^ (Marques et al., 2014).

In tissue engineering, knowledge of bone nanostructure and interactions between inorganic and organic phases are crucial for the production of biomaterials with structural and functional properties similar to natural bone tissue. Generally, these interactions involve anionic and/or cationic functional groups, which are found in the organic matrix and in turn exhibit strong affinity for either calcium or phosphate ions from the mineral phase of bone. Anionic functional groups, i.e., carboxyl-containing and calcium-binding moieties, including proteins, peptide sequences, single amino acids, and COOH groups, are the most extensively investigated chemical groups, which are considered to be instrumental to improving inorganic and organic phase interaction in synthetic nano-composites for bone regeneration (Farbod et al., 2014).

### Bone Cellular Elements and Bone Remodeling

The majority of bone tissue cells in the organic phase are osteoblasts and osteoclasts (Kartsogiannis and Ng, 2004). In particular osteoclasts, cells that derive from the myeloid lineage of hematopoietic precursors of bone marrow and specialized in bone resorption (Charles and Aliprantis, 2014) can also circulate in the bloodstream. On the other hand, osteoblasts derived from mesenchymal stem cells (MSCs) in bone marrow, blood and also from pericytes are involved in bone formation and replace bone removed by osteoclasts (Sims and Civitelli, 2014). It has been reported that MSCs migration to the bone surface is a significant step in bone formation and fracture healing. Indeed, alterations in MSC migration can lead to abnormal bone imbalances. However, MSC migration is a complex mechanism, whose regulation system is yet to be elucidated (Su et al., 2018). Moreover, other osteoblast-derived cells reside in bone tissue, such as bone lining cells and osteocytes. Bone lining cells cover the bone surface, where bone resorption or bone formation is not requested (Miller et al., 1989) while osteocytes derived from osteoblasts suspend their activity when buried in the matrix. It has been suggested that this mechanism represents a form of stress sensor (van Oers et al., 2015). When considered together, these cells are organized into temporary anatomical structures called basic multicellular units (BMUs). BMUs are key grouping cells that carry out bone remodeling, a biological process that leads to structural changes and skeletal renewal (Sims and Martin, 2015). Osteocytes recognize old or damaged bone areas and recruit osteoclast precursors at the remodeling site (Lerner et al., 2019). Bone remodeling consists in some sequential steps: (i) initiation, (ii) reversal, and (iii) termination phases. During the initiation phase, osteoclast precursors are recruited and differentiated into mature osteoclasts to allow bone resorption. Osteoclastogenesis requires specific key mediators, such as the macrophage colony-stimulating factor (M-CSF or CSF-1) and the receptor activator of nuclear factor-kB ligand (RANKL or TNFSF11). In particular, M-CSF is produced by osteoblasts and many other cell types; it is necessary for the proliferation of osteoclast precursors, as well as their differentiation and fusion into osteoclasts (Jiao et al., 2015). On the other hand, RANKL binds its receptor RANK, which is localized on the surface of osteoclast precursors to allow fusion, maturation, survival, and osteoclasts activation (Jimi et al., 1999; Levaot et al., 2015). In this contest, some authors have shown that osteocytes are the main source of the RANKL required for osteoclast formation (Xiong et al., 2015). During bone resorption, several factors that lead to MSCs recruitment and differentiation are released through bone remodeling to enable bone formation in the bone marrow microenvironment (Crane and Cao, 2014). The next transient phase, or reversal phase, consists in bone resorption inhibition in addition to osteoblasts recruitment and the subsequent differentiation that leads to bone formation. Osteoblasts can produce a protein called osteoprotegerin (OPG), which is a decoy receptor for RANKL. Thus, this protein prevents RANKL from binding to RANK, with the consequent inhibiting of osteoclast differentiation and activation (Boyce, 2013). The final step in the remodeling cycle is represented by the termination phase, when an equal amount of resorbed bone has been replaced (Raggatt and Partridge, 2010). Osteocytes contribute to ending the remodeling process by producing sclerostin, which inhibits the bone formation induced by Wnt signaling in osteoblasts (van Bezooijen et al., 2005). At the end of the process, mature osteoblasts undergo apoptosis, become bone lining cells or differentiate into osteocytes (Sims and Martin, 2015). Human skeleton integrity is maintained by a delicate balance between bone resorption and bone formation. Alterations to this mechanism result in several skeletal diseases, such as osteoporosis (McClung, 2018), due to excessive bone resorption, or osteopetrosis caused by excessive bone formation (Sobacchi et al., 2013).

## Stem Cells in Tissue Engineering

In regenerative medicine, stem cells/progenitor cells should have the following important characteristics: (i) availability in large amounts, (ii) multiple differentiation, (iii) painless isolation methods, (iv) use in autologous or allogeneic transplant, (v) agreement with Good Manufacturing Practice guidelines (GMP) (Trohatou and Roubelakis, 2017).

Interestingly, in recent years, many researchers have focused their attention on the analysis of stem cells secretome. Indeed, different reports have been published demonstrating that significant biological functions, such as proliferation, differentiation, communication and migration can be regulated by cellular secreted molecules (Makridakis et al., 2013). Indeed, in clinical practice, MSC secreted molecules may direct different mature cells to differentiate. This differentiation process occurs in specific conditions, such as in the presence of medium composition and/or biomaterials proprieties (Trávníčková and Bačáková, 2018). To this end, biomaterials should be biocompatible, biodegradable and osteoinductive/osteoconductive to allow cellular proliferation and osteogenic differentiation in the healing site (Liu et al., 2010; Perez et al., 2018). Several biomaterials, inspired to facilitate bone composition and structure have been developed and employed in tissue engineering for bone repair (Fillingham and Jacobs, 2016), as reported for ceramics, polymers, and composite scaffolds (Iaquinta et al., 2019).

The presence of stem cells was first reported on in bone marrow (Drela et al., 2019). At present, several types of stem cells have been put forward as a source of osteoblast progenitors (Mushahary et al., 2018). Examples are human embryonic stem cells (hESCs), induced pluripotent stem cells (iPSCs), and human mesenchymal stem cells (hMSCs) (Perez et al., 2018). hESCs *in vitro* cultures were first established in Thomson et al. (1998). hESCs are pluripotent human embryonic stem cells derived from human blastocysts (Kwon et al., 2018). These cells maintain developmental potential for all three embryonic germ layers (endoderm, mesoderm, and ectoderm) even after months of proliferation *in vitro*, differentiating into specific cell types by controlling culture conditions (Thomson et al., 1998). hESC isolation requires human embryo destruction. For this reason, the use of hESCs is considered highly objectionable (Johnson, 2008). Indeed, in many countries, a ban on hESCs has negatively affected hESC research progress, as many governments around the world have not supported research funding. Nevertheless, in certain counties some progress has been made in isolating, culturing, and characterizing hESCs using different strategies (Khan et al., 2018). In order to circumvent ethical issues, proposals have been made to isolate hESCs from a single blastomere, without destroying the human embryo. This goal can be reached using a technique similar to that employed in pre-implantation genetic diagnosis (Chung et al., 2008). Several studies have reported on hESC proliferation and osteogenic compatibility with different biomaterials (Chen et al., 2018). Tang et al. (2012), evaluated the behavior of hESCs *in vitro* when associated with calcium phosphate cement (CPC) showing good cell viability and hESC osteogenic differentiation. Moreover, Liu and his collaborators have studied hESCs seeded onto macroporus CPC for bone regeneration *in vivo* in critical-sized cranial defects in rats (Liu et al., 2014). Similarly, Kim et al. (2008), have shown that hESCs in association with poly (D,L-lactic-co-glycolic acid)/hydroxylapatite composite scaffolds can be used for bone regeneration *in vivo*. However, major drawbacks in the use of hESCs include these significant matters: (i) potential unexpected differentiation, (ii) putative teratoma formation, (iii) culture conditions set up, (iv) immune reactions and (v) the ethical and religious debate (Cunningham et al., 2012). In this context, Takahashi and Yamanaka have developed iPSCs through the use of lentivirus containing four transcription factors (c-Myc, Oct3/4, Sox2, and Klf4), which induced a pluripotent state comparable to hESC (Takahashi and Yamanaka, 2006). Specifically, iPSC reprogramed cells, derived from adult somatic cells as skin fibroblasts, have the ability to re-differentiate virtually into any cell type (Perez et al., 2018). iPSCs show some advantages as a result of (i) by-passing the use of human embryos, (ii) showing morphology and growth properties specific to embryonic cells, (iii) expressing the same hESC marker genes, whereas (iv) these cells can be transplanted into the same patient without the adverse effects of the immune rejection (Takahashi and Yamanaka, 2006; Fu, 2014) as a result of being autologous. In tissue engineering, hiPSCs represent an interesting cell source since patient- or disease-specific mesenchymal/monocyte/macrophage precursors can be generated, which may differentiate into osteoblasts or osteoclasts, respectively (Lou, 2015). Ji et al. (2016), have established that hiPSCs from human gingival fibroblasts isolated from discarded gingival tissues combined with nano-hydroxylapatite/chitosan/gelatine scaffolds could be a potential innovative approach for bone tissue engineering. Moreover, Jeon et al. (2016), have shown that hiPSC-mesenchymal stem cells and macrophages differentiated into osteoblasts and osteoclasts, respectively, when co-cultured on hydroxylapatite-coated poly(lactic-co-glycolic acid)/poly(L-lactic acid) scaffolds. In another study, conducted by Xie et al. (2016) a biomimetic hydroxylapatite/collagen/chitosan (HAp/Col/CTS) scaffold was employed to induce osteogenic differentiation in iPSCs *in vivo*. Their data have shown that combined system iPSCs-HAp/Col/CTS can be used to create personalized and efficacious bone regeneration (Xie et al., 2016).

This review intends to highlight MSCs involvement in regenerative medicine and their potential application with or without scaffolds in clinical practice.

### Mesenchymal Stem Cells

Mesenchymal stem cells are “fibroblastic-like” cells, which form clusters defined as fibroblast-colony forming units (CFU-F) (Friedenstein et al., 1970). MSCs are adherent cells positive for CD73, CD90, and CD105 markers (>95%) and negative for other specific antigens, such as CD45, CD34, CD14, CD79, and HLA class II (<2%) as defined by the International Society for Cellular Therapy (ISCT) (Dominici et al., 2006). MSCs can renew themselves through cell division, whereas they differentiate into osteoblasts, adipocytes, and chondrocytes after exposure to specific soluble factors in the microenvironment (Pittenger et al., 1999; Manfrini et al., 2013; Fitzsimmons et al., 2018). *In vitro*, osteogenic differentiation of stem cells typically involves the use of dexamethasone, β-glycerolphosphate, and ascorbic acid (Manfrini et al., 2013). Moreover, MSCs seem to have potent anti-inflammatory and immunomodulatory properties, in addition to their ability to form cartilage and bone. As a result of these characteristics, MSCs could be employed for the treatment of autoimmune diseases like rheumatoid arthritis, whereas further clinical studies are needed to produce sound evidence (Ansboro et al., 2017; Rotondo et al., 2017).

The ISCT criteria do not provide information about MSCs potential as therapeutic cell sources. Therefore, different comparative studies have been carried out to evaluate the potential of MSCs from various origins in order to select the best source for cell-based therapy (Jin et al., 2013). MSCs can be obtained from different tissues, such as amniotic fluid (AF-MSCs), dental pulp tissues (DPSCs), placental-derived MSCs (PD-MSCs), bone marrow (BM-MSCs) and adipose tissues (ADSCs) (Ullah et al., 2015). It has been reported that about 90% of AF-MSCs express some ESC markers, such as octamer-binding transcription factor 4 (Oct-4). Thus, AF-MSCs can be considered an intermediate stage between embryonic and adult stem cells (Rossi et al., 2014; Markmee et al., 2017). Osteogenic differentiation of AF-MSCs leads to Wnt signaling pathway activation, while Wnt signaling inhibition through selective inhibitor Dickkopf-1 (DKK-1) promotes adipogenesis (D’Alimonte et al., 2013).

Dental pulp tissues were reported to be the first human dental MSCs identified from pulp tissues (Pisciotta et al., 2015). Subsequently, other dental MSCs have been discovered, deriving from human exfoliated deciduous teeth (SHED), tooth germ progenitor cells (TGPCs), dental follicle progenitor cells (DFPCs), periodontal ligament (PDLSCs), alveolar bone-derived MSCs (ABMSCs), apical papilla (SCAP), and gingival MSCs (GMSCs) (Liu et al., 2015). These MSCs, derived from dental tissues, show some proprieties, such as self-renewal, multi-differentiation potential, immunomodulatory functions, as well as an effective capacity for tissue regeneration, including bone tissue (Liu et al., 2015).

The osteogenic differentiation capacity of MSCs derived from placental tissues, i.e., amniotic membrane MSCs (AM-MSCs), umbilical cord MSCs (UC-MSCs), chorionic membrane MSCs (CM-MSCs) and deciduas MSCs (DC-MSCs) have also been studied (Shen et al., 2019). These data have demonstratedthat AM-MSCs and UC-MSCs contain higher osteogenic potential and, therefore, are good sources for bone reconstruction tissue engineering (Shen et al., 2019). Other recent studies have reported that UC-MSCs, deriving from Wharton’s jelly region, show better osteogenic differentiation than other cordon regions (Mennan et al., 2013), while another research has compared UC-MSCs derived from Wharton’s jelly with ADSCs, demonstrating that ADSCs have a higher osteogenic differentiation capacity compared to UC-MSCs derived from Wharton’s jelly after 21 days of osteogenic differentiation (Zajdel et al., 2017).

Adipose tissues and BM-MSCs are probably the most common MSCs used in clinical practice (Fitzsimmons et al., 2018). Donor characteristics, such as age or body weight, can play a role in both the quality and quantity of collected MSCs (Marędziak et al., 2016). Specifically, it has been reported that BM-MSCs show altered proliferation and senescence with increasing age, while ADSCs do not demonstrate these negative age-related effects (Zhu et al., 2008; Beane et al., 2014).

It has been reported that biomarker levels in relation to senescence at ADSCs and BM-MSCs passages 6 and 10, such as SA-gal activity and p21 gene expression, were lower in ADSCs compared to BM-MSCs, which may contribute in part to the higher proliferation rate and differentiation potential of ADSCs (Chen et al., 2012).

On the other hand, Choudhery et al. (2014), showed that ADSC functions are influenced by advancing age. Furthermore, it has been reported (Liu et al., 2018) that transfected-BM-MSCs with microRNA miR-26a, which were previously investigated by Luzi et al. (2012) as a possible target for bone disease RNA-based therapy, improved the bone repair process of cranial bone defects in mice. Moreover, some MSC factors have been shown to be able to influence the differentiation abilities of these cells (Wang et al., 2015). It has been reported that UC-MSCs secretion factors can initiate osteogenesis of BM-MSCs in rat calvarial bone critical defects (Wang et al., 2015).

Mesenchymal stem cells substantially can be obtained from almost any tissue of the human body. However, stem cells collecting process and donor characteristics can represent practical drawbacks. For these reasons, the operator must consider the difficulty in obtaining samples and the potential adverse effects in collecting the cells from the donor in order to select an adequate cell source (Vishnubalaji et al., 2012). For example, collecting BM-MSCs from the donor can be painful, or resulting in bleeding and infection. On the other hand, ADSCs are abundant, while representing one of the main stem cell source. In addition, collecting ADSCs is much less painful procedure compared to other stem cell sources (Zare et al., 2018).

### Mesenchymal Stem Cells in Cell Therapies

In the bone regeneration field, cell-based therapies using MSCs can provide solutions to several problems relating to bone fractures due to trauma or bone diseases (Grayson et al., 2015). When bone is subjected to inflammatory stimuli, a cascade of inflammatory and regenerative events occur to allow local repair and bone healing (Loi et al., 2016). This process includes some sequential events, such as the local and systemic release of pro-inflammatory cytokines, recruitment of immune cells to the damaged site, soft-tissue inflammation and edema, differentiation of osteogenic progenitor cells, the local release of bone morphogenetic proteins, callus formation and bone remodeling (Grayson et al., 2015). During bone remodeling, MSCs are known to differentiate into osteoblasts to enable bone formation (Crane and Cao, 2014). Endogenous or exogenous MSCs migration to the bone injury site is a crucial step in treating bone disease (Su et al., 2018). In particular, endogenous MSC recruitment is influenced by inflammatory mediators secreted by immune cells (Ren et al., 2010; Almeida et al., 2012), TGF-β1 released by the bone matrix (Wan et al., 2012) or chemokines, such as stromal cell-derived factor 1 (SDF-1 also known as CXCL12) (Kitaori et al., 2009). It has been shown that CXCL12 is found at high levels both in human MSCs and primary osteoblasts; CXCL12 seems to be regulated by Slug, a member of a superfamily of zinc-finger transcription factors required for osteoblast differentiation (Piva et al., 2011). In addition, other chemotactic factors are involved in this process, such as cytokines (e.g., IL-6, TNF-a, and IL-1b) and growth factors (IGF-1, PDGF-BB, TGF-β, and HGF) (Li and Jiang, 2011).

Immediate use of exogenous MSCs after acute injury leads to a decrease in local and systemic inflammatory responses (Grayson et al., 2015). Many studies have reported that MSCs can regulate immune systems by suppressing T cells, reducing activation and proliferation of B-cell and NK cells, while promoting regulatory T cell generation (Yagi et al., 2010; Gao et al., 2014). In contrast, MSCs administrated in intermediate periods after injury, participate in bone repair due to differentiation into chondrocytes and osteoblasts, thus stimulating local endogenous osteoprogenitor cell recruitment (Grayson et al., 2015).

In MSCs-based therapy, a potential limitation is that MSCs do not persist following infusion. Some authors have sustained that a more active immunological process is also responsible for the limited persistence of allo-MSCs. Indeed, Ankrum et al. (2014), have supported the idea that MSCs are “immune evasive” and not “immune privileged,” as defined by others authors (Paterson et al., 2014), since allogenic MSCs from donors may cause immune rejection (Ankrum et al., 2014). The ISCT defined human MSCs as MHC I positive and MHC II negative (Dominici et al., 2006); Le Blanc et al. (2003), have demonstrated that undifferentiated MSCs express low levels of MHC class I and are negative for MHC class II, while differentiated MSCs or MSCs exposed to IFN-γ can express significantly more MHC I and MHC II.

**FIGURE 2:**
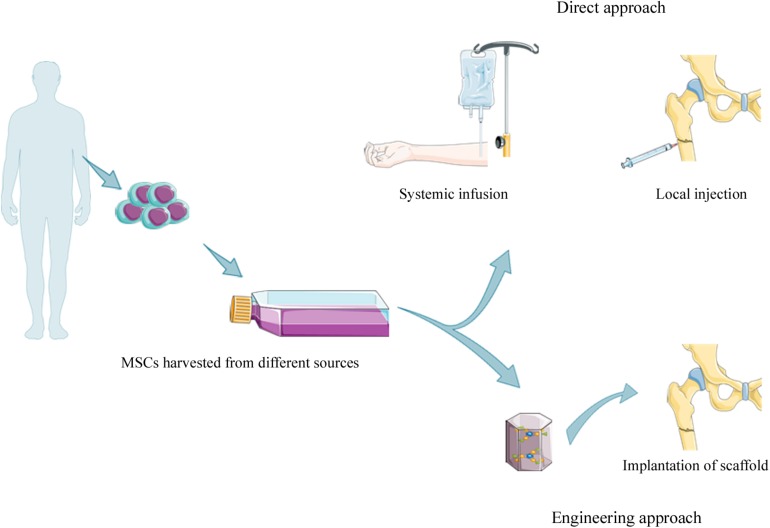
Strategies for MSCs based therapy. MSCs can be isolated from different sources [e.g., amniotic fluid (AF-MSCs), dental pulp tissue (DPSCs), placental-derived MSCs (PD-MSCs), bone marrow tissue (BM-MSCs), and adipose tissue (ADSCs)] with or without culture expansion before clinical application. MSCs can be introduced intravenously by systemic infusion or local injection into fracture site (direct approach), or loaded on scaffold (e.g., ceramics, polymers, and composite) before the implantation into damaged area.

Mesenchymal stem cells harvested from specific tissue in cell therapies, can be utilized with or without culture expansion (Verboket et al., 2018). Moreover, MSCs can be delivered to the injured area of the bone through: (i) systematic or local injections and (ii) engineering techniques (Oryan et al., 2017) (Figure 2).

#### Direct Injection Approach

The direct injection approach includes systemic MSCs administration through intravascular injection (e.g., intravenous and intra-arterial injection). This method enables MSCs to be widely distributed throughout the body using specific cell numbers defined as the effective cell dose (ECD), which is the minimum cell number required to observe a therapeutic effect (Horwitz et al., 2001). However, some limitations exist relating to the formation of microemboli (Boltze et al., 2015). Specifically, since MSC diameters are large (ranging from 15 to 30 μm), MSCs could be trapped in small vessels causing the “first pass effect,” for example, in lungs (Fischer et al., 2009).

The dynamic distribution of MSCs administrated through intra-artery, intravenous and intraperitoneal cavity infusions has been monitored by real-time imaging, immediately after infusion and at 48 h post-infusion in rat. The results showed that MSCs have been detected in the first phase in lungs and then in the liver and other organs, including long bones (Gao et al., 2001). Many researchers suppose that local injection of MSCs is more effective than systemic injections since all MSCs are lost when they are trapped in the lungs after systemic infusion (Oryan et al., 2017).

Direct injection of MSCs involves the bone marrow aspiration mainly from the iliac crest. The aspirated bone marrow, containing many MSCs, is called bone marrow aspirate concentrate (BMAC) (Qin et al., 2014). BMAC is usually reduced in volume to increase MSCs content. During this process red cells and plasma are removed before BMAC injection into damaged areas (Hernigou and Beaujean, 2002). Hernigou et al. (2005), suggested that autologous MSCs could be used for treating fractures in patients with atrophic non-union of the tibia through percutaneous injection of BMAC. A recent study has reported that BM-MSCs injection on day 7 after fracture can improve bone healing in a murine model (Wang et al., 2018). Huang et al. (2015), have demonstrated that systemic and local administration of allogenic BM-MSCs can improve callus formation in fracture healing in rats.

In conclusion, systemic MSCs injection is useful for treating injuries present at multiple sites. Some limitations/problems may arise in patients because of possible microemboli formation. Local MSCs injection is a non-invasive procedure, which is more advisable for a single injury than complex fractures (Raynaud and Rafii, 2013; Abazari et al., 2019).

#### MSCs, Biomaterials, Growth Factors in the Tissue Engineering Approach

Defects less than 50 mm in length might be repaired with autologous bone grafting, while this procedure is inefficient in larger defects (Dumic-Cule et al., 2015). As a result, alternative tissue-engineering strategies combining biomaterials/scaffolds, MSCs and growth factors are used in order to improve bone repair in fractures greater than 50 mm (Decambron et al., 2017). Scaffolds are considered structures, which improve cellular adhesion, proliferation and osteogenic differentiation. Another important characteristic of scaffolds concerns interconnected porosity (the optimal pore size is 200–350 μm) to enable the successful diffusion of nutrients, oxygen, and cellular waste products (Murphy et al., 2010).

It has been established that ECM contains growth factors and cytokines. For these characteristics ECM was employed, as potential therapeutic biomaterial, to promote cell proliferation and differentiation (Frantz et al., 2010). The excised tissue must undergo decellularization. This procedure, which is a combination of physical stress and chemical/enzymatic treatments, allows to remove cells without destroying essential ECM components. Then, decellularized extracellular matrix (dECM) can be used for therapeutic applications. It should be recalled that the composition and spatial orientation of ECM varies from tissue to tissue (Badylak et al., 2015). As mentioned above, bone ECM consists of an organic phase with types I, II, V collagen and non-collagenous proteins. The organic phase constitutes approximately 20% of bone mass, together with the mineral phase. Moreover, bone ECM also contains pro-inflammatory cytokines and several growth factors, such as bone morphogenic proteins (BMPs), vascular endothelial growth factor (VEGF), transforming growth factor β(TGF-β), platelet-derived growth factor (PDGF), and fibroblast growth factors (FGFs) (Papadimitropoulos et al., 2015).

The dECM can be processed for different tissue engineering applications. Thus, dECM can be used as scaffold in order to maintain its original geometry, as bio-ink (see below) or hydrogels (Kim et al., 2019). A recent study reported that tissue-specific hydrogels derived from decellularized bovine bone extracellular matrix (bECM) owns specific mechanical and biological characteristics, including osteogenic potential for clinical use (Sawkins et al., 2013). Moreover, in other investigations, dealing with bone tissue engineering, reported that the combination of bECM hydrogels with DPSCs, which is a source of potential stem cells, is sufficient to induce their osteogenic differentiation (Tatullo et al., 2015) without requiring additional osteogenic factors (Paduano et al., 2017). Thus, dECM is able to mimic in full the complex interactions that take place within the tissue. In addition, dECM because the cellular DNA is almost completely removed during the decellularization process has a lower risk of activating the immune response (Kim et al., 2019). However, one of the main dECM limitation is the lack of standardized procedures. Tissue sources and storage conditions employed before decellularization may influence the quality of dECM, resulting in batch-to-batch differences even within the same tissue type (Kim et al., 2019). To promote bone regeneration, limitations could be circumvented employing also synthetic bone graft substitutes that present specific physic-chemical properties (Ciocca et al., 2017).

Materials employed for bone repair include (i) metals and metal alloys, such as cobalt-chromium, zirconium, titanium, (ii) ceramics and bioactive glasses, which include calcium phosphate (CaP), tricalcium phosphate (TCP) and hydroxylapatite (HA)-derived scaffolds, (iii) biological materials, e.g., collagen and chitosan or synthetic polymers, including polylactic acid (PLA), polyglycolide (PGA) and the copolymer of poly-(DL-lactic-co-glycolic-acid) (PLGA), (iv) composite materials derived from the combination of polymer and ceramic scaffolds, such as HA-collagen scaffolds (Iaquinta et al., 2019).

To date, novel bioactive nanomaterials and nanofabrication techniques allow to control the physical and structural characteristics of new scaffolds (Cross et al., 2016). Indeed, it has been reported that two-dimensional (2D) synthetic nanosilicates (Laponite, Na^+^_0__.__7_[(Mg_5__.__5_Li_0__.__3_)Si_8_O_20_(OH)_4_]^–^_0__.__7_) induce hMSCs to the osteogenic differentiation through dissolution products, such as Na^+^, Mg^2+^, Li^+^, and Si(OH)_4_ (Gaharwar et al., 2013). It has been demonstrated that the incorporation of nanosilicates can improve physical integrity with an increase of the scaffold mechanical strength without any osteogenic supplements (Kerativitayanan et al., 2017).

Overall, it is difficult to create the complex structure of scaffolds with precise conventional techniques. A large variety of methods, i.e., solvent casting/particulate leaching, gas foaming, phase separation or electrospinning, have been used in the fabrication of 3D scaffolds, either as a single procedure or in combination (Turnbull et al., 2018). Moreover, 3D printing is a valid alternative technique developed for the production of scaffolds. Indeed, 3D printing allows to produce scaffolds, layer-by-layer, using powder, liquid or solid material substrates (Mironov, 2003). The microstructure of a 3D printed-scaffold can be obtained by a computer-aided design (CAD) model loaded onto a 3D printer (Do et al., 2015). Bioprinting is a 3D technique employed for the realization of constructs by depositing biological elements, such as cells and growth factors, in order to repair or replace damaged tissues (Skardal and Atala, 2015). To this purpose, bioprinting requires (i) the bio-ink, containing scaffolds in which biological components are encapsulated and (ii) a 3D bio-plotter, which is a 3D printer system used to extrude the bio-ink. Cells for printing can be obtained from tissue biopsies, blood samples or other sources, then expanded *in vitro* to maximize cell density on bioprinting. Cells are encapsulated within the biomaterial to realize the 3D biological construct to be implanted *in vivo*. The interface between cells and scaffolds has a crucial role in tissue regeneration, which is a complex and dynamic microenvironment (Murphy et al., 2014). The different properties of scaffold, such as stiffness and nanostructure, can affect stem cell responses, while enhancing the osteogenic differentiation for bone repair (Zhang et al., 2018). Many efforts have been carried out to design “smart” scaffolds with specific physical/chemical properties incorporating bioactive molecules and nanoparticles, such as growth factors or extracellular matrix (ECM)-like molecules (Motamedian et al., 2015). This approach improves the interactions with cells thus enhancing bone regeneration. Growth factors play a significant role in bone repair/regrowth. Several growth factors and biomolecules control the new bone formation and the ECM deposition. Indeed, growth factors, such as BMPs, VEGF, TGF-β, PDGF, IGF-1, and FGFs have frequently been included in scaffolds (Iaquinta et al., 2019). In regenerative medicine, platelet-rich plasma (PRP) derived from blood plasma and its derivatives are employed to potentiate stem cell proliferation, migration, and differentiation (Santos et al., 2018). However, PRP is not considered to be osteoinductive, whereas the addition of PRP to specific bone graft substitutes can improve the bone healing process (Malhotra et al., 2013). For example, PRP and autologous BM-MSCs synergism when seeded onto macroporous CPC can promote bone regeneration in mini pigs (Qiu et al., 2018).

**FIGURE 3:**
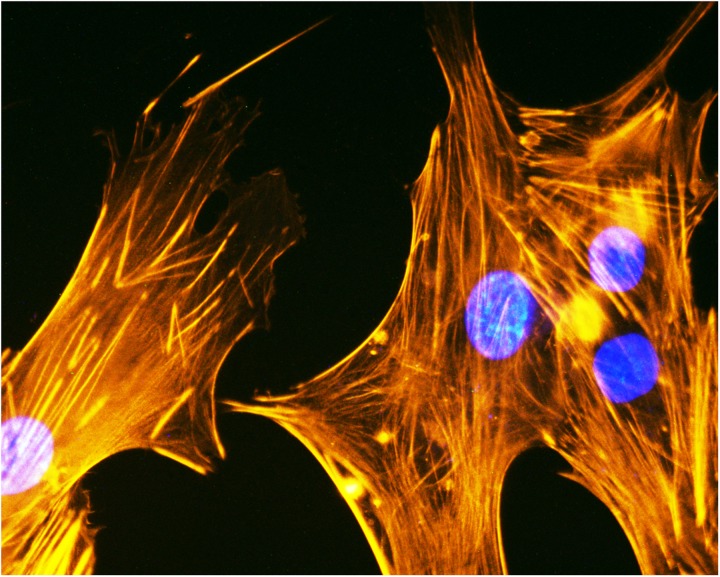
Cytoskeleton analysis of human ADSCs. Cytoskeleton analysis by phalloidin TRITC (tetramethylrhodamineisothiocyanate) staining of human ADSCs grown on the biomaterial (magnification 40x). Cellular nuclei were stained with 0.5 mg/ml DAPI.

In a recent study, the regenerative potential of two MSCs, i.e., AF-MSCs and BM-MSCs were compared *in vitro* and *in vivo* (Mohammed et al., 2019). This research demonstrated that AF-MSCs loaded on gel-foam scaffolds performed better during *in vivo* bone healing than BM-MSCs (Mohammed et al., 2019). Osteogenic differentiation of human ADSCs (Figure 3) loaded onto HA/type I collagen scaffold (Coll/Pro Osten 200^®^), a biomaterial used in maxillofacial surgery for zygomatic augmentation (D’Agostino et al., 2016), was tested *in vitro* to evaluate the expression of specific genes involved in osteogenic differentiation (e.g., SP7 and ALP), as well as adhesion molecules gene expression, such as ECM (Mazzoni et al., 2017a, 2019).

In addition to human ADSCs, engineered human osteoblast-like cells, Saos-eGFP, were employed to evaluate the biocompatibility and bioactivity of HA/collagen-derived scaffolding *in vitro* (Manfrini et al., 2015). Interestingly, it has been reported that HA-derived scaffolding co-doped with gallium, magnesium, and carbonate showed osteogenic and antibacterial abilities. Specifically, doping with gallium can induce antibacterial effects without negative consequences for human ADSCs viability *in vitro* (Ballardini et al., 2018). Further stimulating work has reported that autologous ADSCs, when harvested in accordance with GMP guidelines, were employed to treat 13 cases of cranio-maxillofacial hard-tissue defects (Sándor et al., 2014). These defects were repaired with ADSCs seeded onto bioactive glass or β-TCP scaffolds and, in some cases, with additional recombinant bone morphogenetic protein-2 (BMP-2). Clinical evaluation showed successful integration of the constructs in 10 out of 13 cases (Sándor et al., 2014). A recent study *in vitro* compared ADSCs and BMSCs osteogenic capabilities when seeded onto Bioglass-based scaffolds. Data showed that both ADSCs and BMSCs have similar characteristics, whereas ADSCs seeded onto Bioglass-based scaffolds can differentiate into osteogenic lineage without the use of an osteogenic medium, compared to BMSCs (Rath et al., 2016). On the other hand, another study has revealed that BMSCs seeded onto nanocomposite bioactive glass/gelatine scaffold had higher osteogenesis capacities than UC-MSCs and ADSCs both *in vitro* and *in vivo* (Kargozar et al., 2018). An alternative approach to scaffold-based tissue engineering is the so called “cell sheet” technique, which was used for the first time in 1970 to create tissue from cultured cells (Green et al., 1979). This technique was based on cell sheets derived from hyperconfluent cell cultures characterized by extensive cell-to-cell interaction and its own ECM (Nakao et al., 2019). In addition, the cell sheet can be detached using a temperature-responsive culture dish grafted with a poly(*N*-isopropylacrylamide) in order to preserve cell–cell interactions (Kwon et al., 2018). Indeed, Kushida et al. (2000), have described that MSCs seeded onto temperature-responsive culture dishes can be harvested by reducing the temperature without enzymatic digestion. In tissue engineering, MSC sheets have been used for regenerating different types of organs/tissues including bone tissue, as recently described by Ueyama et al. (2016). Long et al. (2014), have reported that MSC sheets show improved osteogenicity inducing prolonged cartilage and callus formation during critical-sized bone defect repair in mice. Finally, in a pioneering study, Kim et al. (2016), associated the benefits of MSC sheets (derived from canine adipose-derived MSCs) with composite polymer/ceramic scaffolds, such as poly-ε-caprolactone (PCL)/β-tricalcium phosphate (β-TCP). Their results have shown that MSC sheets combined with composite scaffold strongly stimulate and accelerate new bone formation in a critical-sized bone defect *in vitro* (Kim et al., 2016). Additionally, in engineering tissue genetically modified-MSCs which express specific proteins, radioisotopes or microRNAs can be used as anti-tumor vectors owing to their ability to migrate to sites of active primary or meta-static cancers (Belmar-Lopez et al., 2013). Moreover, it is possible to induce modified-MSCs to produce osteogenic and angiogenic growth factors to promote bone regeneration (Nauth et al., 2010).

### Clinical Trials

Mesenchymal stem cells have been studied in a large variety of animal species (e.g., sheep or rabbit) (Gallego et al., 2015; Erdogan et al., 2016). Thus, animal models can be used for understanding mechanisms and applications in clinical settings (Oryan et al., 2017). The clinical trials, which have been made available in the public domain, obtained from the ClinicalTrials.gov database^1^ (June 2019), are listed in Table 1 in order to show the current status of clinical MSC therapy for bone repair.

**TABLE 1:** Clinical trials using MSCs to bone fractures repair.

**NCT number**	**Title**	**Status**	**Conditions**	**Interventions**
NCT02140528	Allogeneic Mesenchymal Stem Cell Transplantation in Tibial Closed Diaphyseal Fractures	Completed	Tibial fracture	Biological: mesenchymal stem cell injection| biological: placebo
NCT01788059	The Efficacy of Mesenchymal Stem Cells for Stimulate the Union in Treatment of Non-united Tibial and Femoral Fractures in Shahid Kamyab Hospital	Completed	Non-union fracture	Other: injection the mesenchymal stem cell in non-union site
NCT02755922	Bone Regeneration With Mesenchymal Stem Cells	Completed	Mandibular fractures	Biological: application of autologous mesenchymal stem cells
NCT00250302	Autologous Implantation of Mesenchymal Stem Cells for the Treatment of Distal Tibial Fractures	Completed	Tibial fracture	Procedure: autologous mesenchymal stem cells implantation
NCT01206179	Treatment of Non-union of Long Bone Fractures by Autologous Mesenchymal Stem Cell	Completed	Non-union fractures	Biological: cell injection
NCT03325504	A Comparative Study of 2 Doses of BM Autologous H-MSC + Biomaterial vs. Iliac Crest Auto Graft for Bone Healing in Non-Union	Recruiting	Non-union fracture	Biological: cultured mesenchymal stem cells| procedure: autologous iliac crest graft
NCT01532076	Effectiveness of Adipose Tissue Derived Mesenchymal Stem Cells as Osteogenic Component in Composite Grafts	Terminated	Osteoporotic fractures	Procedure: cellularized composite graft augmentation procedure: acellular composite graft augmentation
NCT02177565	Autologous Stem Cell Therapy for Fracture Non-union Healing	Completed	Non-union fractures	Biological: carrier plus *in vitro* expanded autologous BMSCs
NCT01842477	Evaluation of Efficacy and Safety of Autologous MSCs Combined to Biomaterials to Enhance Bone Healing	Completed	Delayed union after fracture of humerus, tibial, or femur	Procedure: implantation of bone substitute plus autologous cultured mesenchymal cells
NCT03905824	The Effectiveness of Adding Allogenic Stem Cells After Traditional Treatment of Osteochondral Lesions of the Talus	Recruiting	Osteochondral fracture of talus	Biological: allogenic stromal mesenchymal cells derived from the umbilical cord| procedure: debridement and microfracture
NCT01409954	Collecting Bone Graft During Spinal Decompression and Posterolateral Lumbar Fusion to Better Define Bone Making Cells	Enrolling by invitation	Pseudarthrosis after fusion or arthrodesis	
NCT01041001	Study to Compare Efficacy and Safety of Cartistem and Microfracture in Patients With Knee Articular Cartilage Injury	Completed	Cartilage injury| osteoarthritis	Biological: cartistem| procedure: microfracture treatment
NCT03856021	Microfracture vs. Microfracture and BMAC for Osteochondral Lesions of the Talus	Enrolling by invitation	Osteochondral lesion of talus	Procedure: microfracture with bone marrow aspirate concentrate| procedure: microfracture
NCT01747681	Results at 10 to 14 Years After Microfracture in the Knee	Completed	Articular chondral defect	Procedure: microfracture
NCT02696876	Synovium Brushing to Augmented Microfracture for Improved Cartilage Repair	Recruiting	Defect of articular cartilage| cartilage injury| osteoarthritis, knee	Device: arthroscopic synovial brushing| procedure: microfracture
NCT01626677	Follow-Up Study of CARTISTEMÂ^®^ Versus Microfracture for the Treatment of Knee Articular Cartilage Injury or Defect	Completed	Degenerative osteoarthritis| defect of articular cartilage	Biological: cartistem| procedure: microfracture
NCT00512434	Percutaneous Autologous Bone-marrow Grafting for Open Tibial Shaft Fracture	Completed	Tibial fractures| fractures, open	Procedure: osteosynthesis
NCT02483364	A Clinical Trial to Assess the Effect of HC-SVT-1001 and HC-SVT-1002 in the Surgical Treatment of Atrophic Pseudarthrosis of Long Bones (Bone Cure)	Recruiting	Pseudoarthrosis	Other: HC-SVT-1001 (initial protocol); HC-SVT-1002 (protocol amendment)
NCT00557635	Osseous Setting Improvement With Co-implantation of Osseous Matrix and Mesenchymal Progenitors Cells From Autologous Bone Marrow	Suspended	Tibia or femur pseudo-arthrosis	Procedure: chirurgical procedure

Currently, there are 966 clinical trials available involving the use of MSCs for the treatment of several pathological conditions such as, respiratory syndrome, autoimmune diseases, or immune system diseases. In this review, firstly we considered all the clinical trials related to “mesenchymal stem cells” and “fractures”; subsequently, those labeled as “unknown status,” “withdrawn,” or “not yet recruiting” were excluded. Thus, 19 clinical trials were identified.

Among these, some clinical trials such as NCT01041001, NCT03856021, and NCT01747681 are related to cartilage engineering rather than bone fracture repair.

Mesenchymal stem cells were administrated in several procedures, such as direct injection or biomaterial implantation. Although many studies have been completed, the major limitation of these clinical trials is the absence of published data. In addition, many trials do not provide enough information about protocol, which would be required in order to reproduce this work in other centers/laboratories.

## Conclusion

Mesenchymal stem cells are attractive candidates for cell-based therapy due to self-renewal, multipotent, immunosuppressive, and homing properties. Similarly, MSCs can regenerate damaged tissue, exert autocrine/paracrine effects and modified-MSCs can delivery therapeutic molecules/genes in bone disease treatment.

To date, the specific mechanisms of MSCs in bone healing are yet to be understood vis-à-vis evaluating their performance on large bone defects and defining the best approaches to be used in clinical practice. In particular, further research is required in order to avoid problems relating to unwanted MSC differentiation. To this purpose, standardized protocols are needed to enable the regulation of MSC growth conditions during *ex vivo* expansion. Other limitations in cell therapy concern ethical issues, possible immunological rejection, the limited amount of available stem cells or variability due to donor-related differences. In addition to MSCs isolation and expansion, another challenge for bone regenerative medicine is MSCs delivery to the bone injury. In this context, several osteoinductive/osteoconductive biomaterials have been employed to provide a 3D environment for MSCs at the site of bone fractures in order to promote MSCs angiogenesis and osteogenic differentiation. This approach can be improved by seeding MSCs on appropriate biomaterials in the presence of specific growth factors, such as BMPs (in particular BMP-2 and BMP-7) or PRP.

Moreover, tissue-derived MSC sheets could be used alone or in combination with different scaffolds in order to accelerate the bone healing process in orthopedic and traumatology cases.

However, despite MSC therapy being an interesting development in tissue engineering, further studies are needed to suggest new MSC therapies for bone repairs due to an absence of data from current completed clinical studies. In the future, further knowledge of MSC potential and the use of new therapeutic strategies could allow improved approaches to bone regeneration/healing to be developed.

## Author Contributions

All authors listed have made a substantial, direct and intellectual contribution to the work, and approved it for publication.

## Conflict of Interest

The authors declare that the research was conducted in the absence of any commercial or financial relationships that could be construed as a potential conflict of interest.

## Footnotes

1 https://clinicaltrials.gov/
